# APOBEC3G and APOBEC3F rarely co-mutate the same HIV genome

**DOI:** 10.1186/1742-4690-9-113

**Published:** 2012-12-20

**Authors:** Diako Ebrahimi, Firoz Anwar, Miles P Davenport

**Affiliations:** 1 Centre for Vascular Research, Faculty of Medicine, University of New South Wales, Sydney, NSW, Australia

**Keywords:** Hypermutated HIV, APOBEC3G, APOBEC3F, Motif representation, G-to-A mutation signature

## Abstract

**Background:**

The human immune proteins APOBEC3G and APOBEC3F (hA3G and hA3F) induce destructive G-to-A changes in the HIV genome, referred to as ‘hypermutation’. These two proteins co-express in human cells, co-localize to mRNA processing bodies and might co-package into HIV virions. Therefore they are expected to also co-mutate the HIV genome. Here we investigate the mutational footprints of hA3G and hA3F in a large population of full genome HIV-1 sequences from naturally infected patients to uniquely identify sequences hypermutated by either or both of these proteins. We develop a method of identification based on the representation of hA3G and hA3F target and product motifs that does not require an alignment to a parental/consensus sequence.

**Results:**

Out of nearly 100 hypermutated HIV-1 sequences only one sequence from the HIV-1 outlier group showed clear signatures of co-mutation by both proteins. The remaining sequences were affected by either hA3G or hA3F.

**Conclusion:**

Using a novel method of identification of HIV sequences hypermutated by the hA3G and hA3F enzymes, we report a very low rate of co-mutation of full-length HIV sequences, and discuss the potential mechanisms underlying this.

## Background

Human apolipoprotein B mRNA-editing enzyme, catalytic polypeptide-like 3G and 3F (hA3G,F) are enzymes important in the control of viral growth [1,2]. They are known for their ability to block HIV infection in the absence of the virion infectivity factor (Vif) [3]. The mechanisms of inhibition proposed for these proteins can be classified as either cytosine deamination-dependent [4] or deamination-independent [5]. In the former, one or more hA3G and/or hA3F molecules are trafficked into a nascent virion and are released along with the viral RNA into the cytoplasm of a newly infected cell. The HIV RNA is reverse transcribed in the host cell to create a DNA minus strand followed by degradation of the original RNA and its replacement by a DNA plus strand. During the degradation of the viral RNA, regions of the DNA minus strand remain transiently unpaired. These single stranded DNA regions are targeted by hA3G and/or hA3F [6]. These enzymes mutate cytosine to uracil within specific sequence motifs. For example hA3G and hA3F preferentially target C within the context of CC and TC, respectively (the underlined cytosine is deaminated to uracil). The uracils in the minus strand pair with adenosines in the plus strand. Therefore the action of hA3G, F results in the replacement of G by A in the plus strand and subsequently in the HIV RNA genome. The G-to-A mutation hotspots are GG and GA for hA3G and hA3F, respectively [7,8]. Usually the HIV sequences targeted by these enzymes show G-to-A mutations in multiple positions; therefore are referred to as hypermutated sequences [9].

It is known that hA3G and hA3F are widely co-expressed in the human cells [10,11] and co-localize in the mRNA processing (P) bodies [12]. They might also co-package into nascent HIV virions, thus acting cooperatively to inhibit HIV infection [10,11]. Despite this, most of the studies so far have concentrated on the impact of individual APOBEC3 proteins. Therefore, the collective impact of these proteins on the HIV genome is not clear. The limited studies of the effect of both proteins have returned contradicting results. Analysis of hypermutated sequences from the HIV-1 pol [13], vpu/env [14], gag [15] and also from the near complete viral genomes [16] points to the substantial domination of either hA3G or hA3F. However, lightly mutated sequences with footprints of both proteins have also been reported [14]. In addition, analysis of fractional *env/nef*[17] and in a different study, vif, gag and env [18] have shown sequences with mutations within both GG and GA motifs. However, given short sequence reads and the limitations of current identification methods, it can often be hard to identify the target motif preferences of hypermutation. The aim of this study is to find out what proportion of the *in vivo* full genome HIV sequences are targeted by both proteins. To achieve this aim the first step is to accurately identify sequences that contain signatures of mutation by hA3G and/or hA3F [7,19]. To investigate the joint impact of hA3G and hA3F, we developed a method that identifies sequences hypermutated by hA3G, hA3F or both by taking advantage of the unique context-dependency of G-to-A mutation by hA3G and hA3F. The target motifs GG (for hA3G) and GA (for hA3F) are less represented in the genomes of hypermutated sequences compared to those of normal HIV sequences. By contrast, the product motifs (AG and AA) are more represented in the hypermutated sequences compared to normal sequences. Therefore, a measure of ‘motif representation’ [20] can be used to identify affected sequences. It is worth noting that all hA3 proteins, except for hA3G, have a dinucleotide target preference similar to that of hA3F [21]. Therefore, any signature attributed to hA3F in this paper could well be the footprint of other hA3 proteins with a GA-to-AA mutation preference. The reason for the use of hA3F here is its greater mutagenic activity against HIV when compared to the other hA3 members with the same motif preference.

Here, we calculate the representation of hA3G and hA3F target and product motifs in 2829 HIV-1 sequences as well as in the 88 full genome HIV-1 sequences classified as “hypermutated” in the Los Alamos National Laboratory (LANL) database. These 2917 sequences are from all groups (M, O, N and P), subtypes (A, B, C, D, F, G, H, J and K) and recombinant forms (e.g. 01_AE, A1D, A2C, A1DK, A1A2D, 21_A2D, 02_AG, 04_cpx, 05_DF, BF and the like) that have been reported in the database. The results showed only one sequence, out of approximately one hundred hypermutated sequences, with clear signatures of mutation by both hA3G and hA3F. Interestingly this sequence does not belong to any of the subtypes and recombinant forms of the HIV main (M) group and has been classified as an outlier (O) HIV-1 sequence. Analysis of *in vitro* hypermutated sequences as well as simulated hypermutation data further confirmed the efficiency of the method based on motif representation in identification of co-mutated sequences.

## Results

### Identification of hypermutated sequences

**Figure 1:**
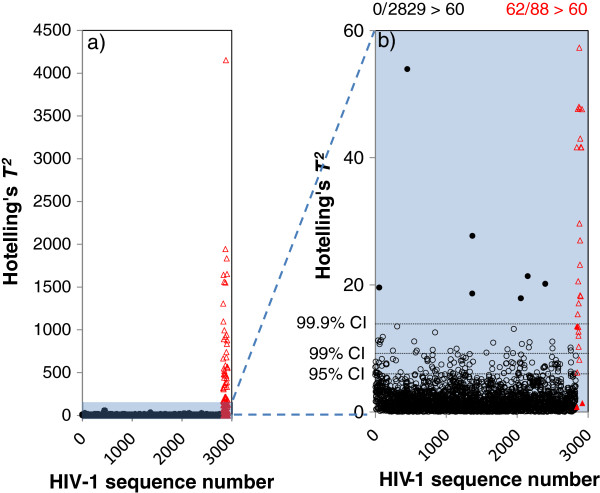
**The Hotelling’s *T***^***2 ***^**statistics of HIV-1 sequences.** There are in total 2917 full genome (> 7000 n.t.) HIV-1 sequences including 2829 nominally normal and 88 nominally hypermutated sequences. The hypermutated sequences are from no. 2830-2917 on the horizontal axis. The filled circles are the nominally normal sequences which are significantly different from the normal HIV-1 population at >> 99.9% confidence levels, thus appear to be hypermutated. The filled triangles are the nominally hypermutated sequences that are significantly different from the normal HIV-1 population at < 50% confidence levels, thus appear to be normal.

The Hotelling’s *T*^*2*^ statistics of the HIV-1 sequences are shown in Figure 1. In this figure, the 2829 sequences that are “normal” according to the LANL database appear first on the horizontal axes (shown by black circles) followed by the 88 sequences identified by LANL as “hypermutated” (shown by red triangles). Figure 1a) shows all the sequences and Figure 1b) the lower range of *T*^2^ axis (*T*^2^<60) that covers all the nominally normal HIV-1 sequences and some of hypermutated sequences. As indicated, compared to normal HIV-1 sequences, the hypermutated sequences exhibit larger *T*^*2*^ values (up to over 800 times larger). The 95%, 99% and 99.9% confidence interval of the Hotelling’s *T*^*2*^ are the values 6.00, 9.23 and 13.85, respectively. Figure 1b shows 7 sequences indicated by filled circles (see Table 1), that are different from the population of normal sequences at confidence levels (α) >> 99.9%. The high significance levels of these sequences may imply that they are hypermutated sequences which have been misclassified as normal in the database.

**Table 1:** **A list of seven HIV-1 sequences with *α*>> 99.9% and two HIV-1 sequences with *α *< 50%**

***Accession Number***	***LANL***	***DR***
AF193275	N	H
FJ469751	N	H
FJ388944	N	H
JF683737	N	H
AY773339	N	Outsider
GU595150	N	H
FJ388965	N	H
FJ900269	H	N or LM
AY169814	H	N or LM

N: Normal; H: Hypermutated; LM: Lightly mutated.

Among those sequences tagged as hypermutated in LANL database, we identified two sequences (shown by filled triangles under the 95% confidence interval line in Figure 1b) that are different from the population of normal HIV-1 sequences at confidence levels less than 50%. This may imply that these two sequences are normal HIV-1 sequences which have been misclassified as hypermutated in the database. Another explanation could be that these are lightly mutated sequences for which it is not possible to discriminate random G-to-A mutation (by reverse transcriptase) [22] from context dependent G-to-A mutation (by hA3G,F) within a large population of HIV sequences. Therefore, they appear within the range of normal HIV-1 sequences. Table 1 shows the accession numbers of the sequences for which we estimate a hypermutation status different from those of the LANL database.

### Identification of source of hypermutation

**Figure 2:**
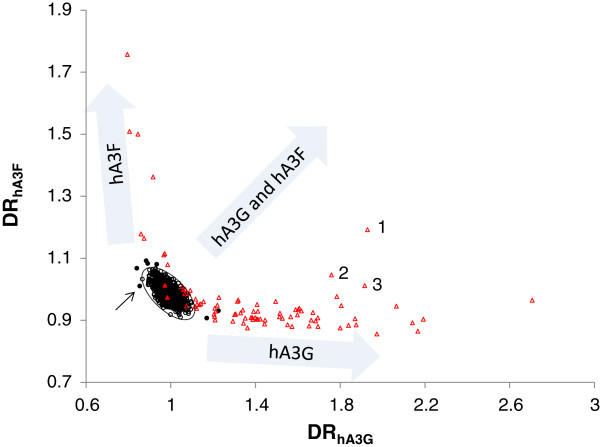
**The plot of DR**_**hA3G**_**versus DR**_**hA3F **_**of 2917 full genome (>7000 n.t.) HIV-1 sequences.** The sequences identified as normal and hypermutated by LANL are shown using black circles and red triangles, respectively. The seven nominally normal sequences with *α* >> 99.9% (appear outside the 99.9% confidence interval broken line) are shown by filled circles. The two nominally hypermutated sequences with *α* < 50% are shown by filled triangles. The sequences hypermutated by hA3G appear along the horizontal axis and those affected by hA3F extend along the vertical axis. The sequences co-mutated by hA3G and hA3F locate between these two groups.

In order to identify which protein (hA3G, hA3F or both) is responsible for hypermutating the HIV sequences a plot of DR_hA3G_ versus DR_hA3F_ (Figure 2) is used here. In this figure the nominally normal and hypermutated sequences are shown by circles and triangles, respectively. The nominally normal sequences with *T*^*2*^ >13.85 which lie outside the 99.9% confidence interval (broken line) are shown by filled circles. The two nominally hypermutated sequence which are very similar to normal sequences (*α*<50%) are shown by filled triangles. As indicated the normal HIV-1 sequences form a tight cluster around ratios at DR_hA3G_ =1 and DR_hA3F_ =1. In this population DR_hA3G_ and DR_hA3F_ are inversely correlated (see the slope of the open circles in Figure 2), presumably due to a general G-to-A mutation error by the HIV reverse transcriptase. This dependency is significantly reduced in the hypermutated sequences in which the G-to-A mutation is motif dependent and different for hA3G and hA3F. These unique features of Figure 2 enable one to identify source(s) of hypermutation.

The following points can be inferred from this figure:

1- Type of hypermutation

The hypermutated sequences are classified into three categories shown by filled arrows. The first category contains sequences with a high DR_hA3G_ which appear along the horizontal axis. These sequences show signs of mutation by hA3G. The second category contains sequences with a high DR_hA3F_ which stretch along the vertical axis. These sequences have footprints of mutation by hA3F. The third category includes sequences which are high in both DR_hA3G_ and DR_hA3F_. These sequences which have footprints of co-mutation by hA3G and hA3F would appear in the top right quarter of the graph. The sequence shown by number “1” is from this category.

In Figure 2 seven nominally normal sequences with *α* >>99.9% are shown with filled circles. Two of these sequences extend in the direction of mutation by hA3G and four in the direction of the hA3F axis, implying that they are hypermutated sequences. There is one sequence (shown by a small arrow) that appears outside the 99.9% interval but does not extend in either direction.

2- Extent of hypermutation

Within the reported sequences in the database, the number of sequences targeted by hA3G is much larger than those affected by hA3F. The extent of hypermutation by hA3G is also greater than that of hA3F as evidenced by the larger scale of the DR_hA3G_ axis compared to that of DR_hA3F_. The calculated values of DR_hA3G_ and DR_hA3F_ can be used as a measure of the extent of mutation. In general the larger these values the more mutation the sequence has undergone.

3- Mutation by both hA3G and hA3F

The sequences which are mutated by both hA3G and hA3F would be located away from the normal sequences and between the sequences targeted by either hA3G or hA3F. The contribution of each protein in hypermutation can be determined from the location of the targeted sequence in the space of DR_hA3G_ versus DR_hA3F_. Surprisingly only one sequence, shown by “1” and belonging to the outlier HIV group, exhibits a clear footprint of both proteins. However it is located closer to the line of hypermutated sequences by hA3G indicating a greater contribution from hA3G. This sequence (accession number AF407419) was isolated from an HIV patient in 1992 and contains multiple stop codons in all open reading frames. There are also two other sequences (shown by numbers 2 and 3) with slightly elevated DR_hA3F_ values compared to those of the remaining sequences mutated by hA3G.

Within the HIV-1 sequences, there is no extensively co-hypermutated sequence with an equal or greater contribution from hA3F. A list of hypermutated sequences at >99.9% confidence level, their accession numbers and source(s) of mutation is given in the Additional file 1: Table S1.

### Analysis of negative strands

Our analysis suggests that motif representation is a useful method for classifying HIV sequences as hypermutated. However, it is also possible that the variations in motif representation we observe arose by random chance, or non-specific mutation. Since these processes are expected to affect the positive and negative HIV strands equally, whereas hypermutation should show its signature only on the positive strand, we therefore analysed the motif representation of the negative strands. Consistent with a strand-specific mechanism of mutation, the motif ratios of the negative strands clustered much more evenly than the positive strands, with no clear evidence for context-dependent mutation. We note also that issues such as codon usage or conserved regulatory elements that may bias motifs should be equally present in negative strands [using the complement of the motif]. This confirms the utility of our method and that it is not excessively biased by these factors. The results are given in Figure S2 of the Additional file 1.

### In vitro hypermutation

**Figure 3:**
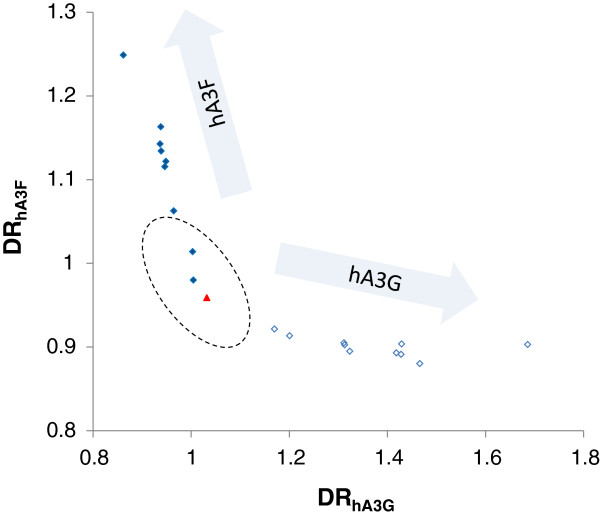
**The plot of DR**_**hA3G **_**versus DR**_**hA3F **_**of a consensus HIV-1 sequence and 19 sequences hypermutated by either hA3G or hA3F.** The consensus sequence is shown using a red triangle. Hypermutated sequences by hA3G or hA3F are shown using open and closed rhombuses, respectively. The 99.9% confidence interval of the subtype B HIV-1 population is shown by an oval. The sequences hypermutated by hA3G appear along the horizontal axis, and those hypermutated by hA3F extend along the vertical axis. There is no co-mutated sequence in this dataset.

In order to confirm the efficacy of the proposed method in identifying co-mutated sequences we analysed a data set consisting of 19 clade B HIV-1 sequences that have been hypermutated *in vitro* by either hA3G or hA3F [7]. This data set does not contain any co-mutated sequence; ten of these sequences have been hypermutated by hA3G, and nine by hA3F (see reference [7] for details). Thus the analysis is expected not to return any HIV-1 sequences with elevated DR_hA3G_ and DR_hA3F._ This is expected to be characterized by an empty space between the axes DR_hA3G_ and DR_hA3F_ in a plot similar to Figure 2. The results of the analysis of these sequences are shown in Figure 3 and Table 2. In Figure 3 the non-hypermutated consensus sequence is shown by a triangle and the hA3G and hA3F hypermutated sequences by open and closed rhombuses, respectively. The pattern displayed in this figure is very similar to the pattern of *in vivo* sequences shown in Figure 2. The *in vitro* sequences hypermutated by hA3G lie on the right hand side of the consensus sequence and extend along the horizontal axes depending on their level of hypermutation. The *in vitro* hypermutated sequences by hA3F locate on the left hand side of the consensus sequence and extend along the vertical axis. The hypermutated HIV-1 sequences (except for two lightly mutated by hA3F) were identified at very high confidence levels (α >> 99.9%). Importantly, no sequence was located in the area between the hA3G and hA3F hypermutated sequence that is characterized by co-mutation.

**Table 2:** **The accession numbers, DRs, *T***^***2 ***^**values and hypermutation status of the HIV-1 sequences hypermutated *in vitro *by hA3G (sequences starting with 3G) or hA3F (sequences starting with 3F)**

**Sequence**	**DR**_**hA3F**_	**DR**_**hA3G**_	***T***^***2***^	**α > 99.9%**
consensus	0.96	1.03	1.85	
3G6	0.89	1.43	190.90	√
3G7	0.90	1.69	559.86	√
3G8	0.92	1.17	28.32	√
3G33	0.88	1.47	224.16	√
3G40	0.90	1.31	98.47	√
3G72	0.91	1.20	39.09	√
3G105	0.89	1.42	182.50	√
3G108	0.89	1.32	103.49	√
3G111	0.90	1.31	98.75	√
3G113	0.90	1.43	200.02	√
3F11	**1.01**	**1.00**	**3.69**	**×**
3F14	1.16	0.94	79.28	√
3F18	1.25	0.86	152.79	√
3F41	1.12	0.95	46.12	√
3F52	1.06	0.96	14.27	√
3F114	**0.98**	**1.00**	**0.20**	**×**
3F116	1.13	0.94	53.23	√
3F117	1.12	0.95	40.87	√
3F124	1.14	0.94	59.93	√

√: Identified with high confidence ×: Not identified with high confidence.

### Analysis of simulated hypermutation

Since neither the analysis of patient sequences from the database nor *in vitro* hypermutated sequences identified co-mutated genomes, this raises the question of whether our method can detect co-mutated sequences if they occur. In the absence of experimental sequences that appear co-mutated, we simulated co-mutation as described below.

**Figure 4:**
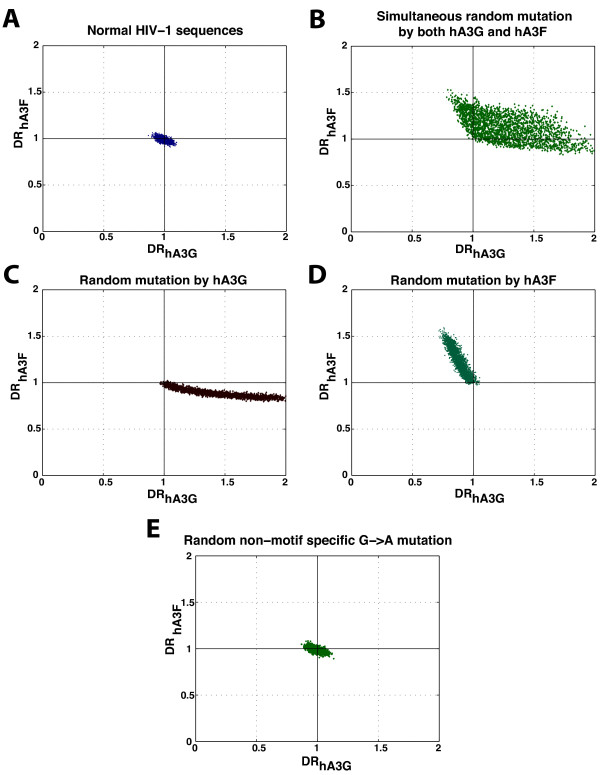
**The plot of DR**_**hA3G **_**versus DR**_**hA3F **_**for A) normal HIV-1 population and simulated mutations by B) both hA3G & hA3F (GG-to-AG & GA-to-AA), C) hA3G (GG-to-AG), D) hA3F (GA-to-AA) and E) reverse transcriptase (random G-to-A).**

We used the normal complete genome HIV-1 population described earlier and randomly mutated 20-200 G nucleotides to A nucleotides either within GG (for hA3G) or within GA (for hA3F), or within both motifs together. For comparison we also performed, in a separate simulation, context-independent (random) G-to-A changes to mimic the effect of reverse transcriptase. The results are shown in Figure 4. As expected the direction of the extension of the HIV sequences in Figure 4 is very similar to those of *in vivo* sequences shown in Figure 2. Mutation by either hA3G or hA3F led to the spread of sequences along the respective axes. When both motifs were targeted by mutation, co-mutated HIV sequences filled the space between the two axes DR_hA3G_ and DR_hA3F_ as expected, whereas this space is almost empty for the *in vivo* data.

The motif dependent G-to-A changes by hA3G and/or hA3F results in an increase in DR_hA3G_ and/or DR_hA3F_, However, random G-to-A changes by reverse transcriptase do not affect these two diagnostic ratios. As a result the HIV population generated by a random (context-independent) G-to-A mutation simulation does not differ from the original HIV population (see panels A and E of Figure 4). The results of the analysis of the simulation data imply that co-mutation by hA3G and hA3F is detectable if present and that its absence from the *in vivo* sequences suggests it is a rare event.

## Discussion

The proteins hA3G and hA3F are important enzymes in controlling viral replication. One of their mechanisms of action is the introduction of multiple mutations into the genome of the affected viruses. The genes for these two proteins are located in close proximity on chromosome 22. They are widely co-expressed in different tissues and also co-packaged into the HIV virions. This may indicate that hA3G and hA3F act cooperatively against HIV. There are many reports on the mutagenicity of each of these individual proteins, but their co-operative action is less understood [11]. This may be partly due to the analysis of short sequences (typically < 10% of the complete HIV genome) [11,13-15,17,23] within which it is not trivial to accurately identify mono- versus co-mutated signatures. Also the previous method of identification has usually been based on alignment and comparison of suspected hypermutated sequences to a consensus sequence which may or may not be the true ancestral sequence of hypermutated sequences. Here, we studied the cooperative impact of hA3G and hA3F by investigating the mutation footprint they have left on the full genome hypermutated HIV sequences. We developed a novel bioinformatics approach for accurate identification of sequences hypermutated by either or both of these two enzymes. Identification of hypermutated sequences is important as they contain information about the mutation mechanism by hA3G and hA3F. Our method identifies hypermutated HIV-1 sequences using the within sequence signature(s) of G-to-A changes by hA3G and/or hA3F. It also uniquely discriminates among G-to-A mutation by reverse transcriptase, hA3G, hA3F and both of these latter proteins. It is based on the representation of hA3G and hA3F target and product motifs in the genome of thousands of HIV sequences. The representation of each motif is quantified in such a way that it becomes independent of the frequency of its constituent mononucleotides. We have coded the algorithm into an executable Java program ‘hypersign’ that is available as an Additional file 2. Analysis of a large population of full genome HIV-1 sequences revealed that co-mutation of the same genome by hA3G and hA3F is a rare event. We performed simulations to confirm that co-mutation is detectable by our method, confirming its absence is not an artifact of the analytic approach.

Two possible mechanisms can be postulated to explain the rarity of co-mutated sequences. Firstly, a low proportion of total sequences showing any hypermutation was observed, suggesting very few virions incorporate APOBEC3 proteins [24]. This could either arise because most virions have no co-packaged APOBEC3, but a few virions package one and some co-package two copies of the enzymes. In this scenario, it is easy to imagine that the number of copies of APOBEC3 should be Poisson distributed. So, assuming random sampling, if 12 out of 2917 genomes (see Figure 2) have at least one hA3F protein and 79 out of 2917 have at least one hA3G, then we would only expect 1×10^-4^ virions having at least one copy of each enzyme. Thus, given the small number of total sequences showing hypermutation, the low frequency of co-mutated sequences maybe explained simply by the low chance of incorporation of either enzyme individually.

Alternatively, hypermutation may arise because Vif fails to exert its effects in a small proportion of cells, leading to co-packaging of many copies of APOBEC3 into the genomes produced from these cells. In this case, we might expect that many genomes that contain either hA3G or hA3F would in fact have copies of both enzymes. In this case, then the lack of co-mutation may suggest either that these two proteins compete for the same HIV RNA, or that co-packaged hA3G and hA3F interfere with the enzymatic activity of one another, perhaps by forming hetero-oligomers [10-12]. An alternative possibility is that, in the presence of co-packaged hA3G and hA3F, one enzyme acting before the other may somehow disrupt the target motifs or inhibit our ability to recognize co-mutation. However, we have simulated these enzymes acting in different orders, and find this does not significantly affect the ability to recognize co-mutated sequences (data not shown); therefore this seems to be an unlikely explanation.

## Conclusion

Our analysis clearly illustrates that co-mutation by hA3G and hA3F is a rare occurrence. However, we are unable to draw conclusions as to the biological mechanisms behind this. Further sequencing of full-length HIV genomes derived from *in vivo* infection might help identify if the frequency of co-mutation is consistent with a Poisson distribution of hA3G and hA3F singly mutated genomes. However, our estimates are that this would require a large number of sequences to accurately estimate this frequency. Alternatively, experimental approaches to determine the accurate stoichiometry of hA3 molecules per virion *in vivo* might also identify whether a low frequency of incorporation and low copy number of incorporated enzymes is present.

## Methods

In order to distinguish hypermutated from normal sequences a measure of the representation of hA3G and hA3F target and product motifs is required. The “representation” of a motif needs to be a quantity that signifies the difference between the observed and expected probabilities of the motif. Simply using the observed probability (relative frequency) of a motif to infer under- or over-representation is inappropriate. This is because the observed probability of a motif is not an independent entity and is influenced by the relative frequencies of its sub-motifs. For example the observed probability of the dinucleotide AA might be very high in a sequence, simply because the sequence has a high proportion of the mononucleotide A. Therefore AA, despite being frequent, is not over-represented. Thus the observed probabilities of sub-motifs need to be considered when estimating the expected probability of a motif.

Representation can be defined as a ratio of observed over expected probabilities. The observed probability (p_obs_) of a motif is the total counts of the motif (e.g. AG) in the sequence divided by the total counts of all other possible motifs with the same length (AA, AC, AG, …, TT). The expected probability (p_exp_) of a motif can be calculated using the observed probabilities of its sub-motifs [20]. For example the expected probability of the dinucleotide AG is the product of the observed probabilities of the mononucleotides A and G. Eq. 1 shows the representation (D) of dinucleotide AG as a typical example.

(1) $$D\left(\mathrm{AG}\right)=\frac{p_{\mathrm{obs}}\left(\mathrm{AG}\right)}{p_{\exp}\left(\mathrm{AG}\right)}=\frac{p_{\mathrm{obs}}\left(\mathrm{AG}\right)}{p_{\mathrm{obs}}\left(A\right)\times p_{\mathrm{obs}}\left(G\right)}$$

The representations of hA3G and/or hA3F target motifs (GG and GA, respectively) decrease and those of product motifs (AG and AA, respectively) increase in hypermutated sequences compared to normal HIV sequences. We define two ratios of product over target representations, one for hA3G (Eq. 2) and one for hA3F (Eq. 3). As will be described later these diagnostic ratios (DRs hereafter) are used together in a bivariate distribution to identify hypermutated sequences.

(2) $$DR_{\mathrm{hA}3G}=\frac{D\left(\mathrm{AG}\right)}{D\left(\mathrm{GG}\right)}$$

(3) $$DR_{\mathrm{hA}3F}=\frac{D\left(\mathrm{AA}\right)}{D\left(\mathrm{GA}\right)}$$

The dinucleotide GG is changed to AG by hA3G; therefore, those HIV sequences that have been targeted by hA3G are expected to have a higher DR_hA3G_ compared to normal sequences. By the same token, mutation by hA3F results in an increase in DR_hA3F_. Sequences that have been affected by both proteins hA3G and hA3F show an increase in both DRs. Importantly we do not measure simply the frequency ratio of AG/GG (for example in the case of hA3G). Rather, we find the ratio of the observed relative frequency of AG to the expected relative frequency of AG (based on the underlying relative frequencies of A and G in the sequence) divided by the ratio of the observed relative frequency of GG to the expected relative frequency, thus accounting for variations in base counts between sequences.

We note that this analysis of dinucleotide motifs can be extended to incorporate longer motifs, and indeed in our previous work we have studied the motif representation of dinucleotides, trinucleotides, and tetranucleotides [20]. However, as the motif preference of hA3F is not found to extend beyond dinucleotide in the full genome *in vivo* HIV-1 sequences (see Figure S1 of the Additional file 1), we utilise only the dinucleotide motif for both enzymes. In addition, although factors such as codon bias and conserved regulatory motifs might affect the absolute value of the representation of motifs at a population level, they do not affect the proposed method that is based on the ‘difference’ in the representation of motifs from normal and hypermutated sequences. That is, we do not require normal sequences to have a ratio of exactly one, but rather empirically determine the ‘normal’ range for the ratio, which includes these factors.

We downloaded 2829 full genome (> 7000 n.t.) HIV-1 sequences from the LANL database as well as 88 sequences identified as “hypermutated” by LANL, in June 2011. For each sequence we calculated DR_hA3G_ and DR_hA3F_ and then the Hotelling’s *T*^*2*^ statistic. The Hotelling’s *T*^*2*^ statistic (Eq. 4) is an extension of the Student *t* statistic to multivariate distributions. It is used to determine group membership in data with more than one measured variable [25].

(4) $$T_{i}^{2}=\left(x_{i}-\bar{x}\right)S^{-1}{\left(x_{i}-\bar{x}\right)}^{\prime }$$

In this work, *x*_*i*_ is a vector of length two containing DR_hA3G_ and DR_hA3F_ of sequence *i*, *x* is a vector of length two containing the two averages of 2829 DR_hA3G_ and DR_hA3F_ from the normal HIV-1 sequences. ***S*** is the variance-covariance matrix.

The Hotelling’s *T*^*2*^ statistic of a given HIV-1 sequence is the square of the Mahalanobis distance of the sequence from the centre of the population of normal HIV-1 sequences in a two-dimensional space specified by DR_hA3G_ and DR_hA3F_. The larger this distance the less likely the sequence is normal, and therefore the more likely it is hypermutated. For each sequence, the likelihood of its membership to the normal HIV-1 population is quantified using the probability associated with its *T*^*2*^ statistic [25]. The confidence level (α) of the Hotelling *T*^*2*^ statistic is given by Eq. 5

(5) $$T_{i}^{2}=\frac{J\left(I-1\right)}{\left(I-J\right)}F\left(\alpha ,J,I-J\right)$$

where *I* is the number of HIV sequences (here 2829), *J* is the number of variables (here two, DR_hA3G_ and DR_hA3F_), *F* is the Fisher’s *F* statistic at the confidence level α and degrees of freedom *J* and *IJ*.

To test the accuracy of the group membership prediction by our proposed method, we performed the same analysis on 19 HIV-1 sequences mutated *in vitro* by hA3G or hA3F [7].

## Competing interests

The authors declare that they have no competing interests.

## Authors’ contributions

DE developed the method, performed the analysis and wrote the manuscript. FA developed the software ‘hypersign’ and performed data analysis. MD supervised the project and revised the manuscript. All authors read and approved the final manuscript.

## Supplementary Material

Additional file 1 **Figure S1.** DR_hA3G_ and DR_hA3F_ of preferred dinucleotide, trinucleotide and tetranucleotide motifs in the normal and hypermutated HIV-1 sequences. **Figure S2.** Analysis of the hA3G and hA3F footprint on the negative strand of the HIV-1 sequences. **Table S1.** Details of the HIV-1 sequences identified as hypermutated at > 99.9% probability level using the proposed method in this paper. **Figure S.** The plot of DR_hA3G_ versus DR_hA3F_ for normal HIV-1 subtypes B, C and A1. Click here for file

Additional file 2 **Hypersign.** A tool for identification of hypermutated sequences. Click here for file
