# Supplementary material for: APOBEC3G and APOBEC3F rarely co-mutate the same HIV genome
Source: Retrovirology. 2012 Dec 20;9:113. doi: 10.1186/1742-4690-9-113 (PMC3532371; doi:10.1186/1742-4690-9-113)
Supplement: Additional file 1 — Figure S1. DRhA3G and DRhA3F of preferred dinucleotide, trinucleotide and tetranucleotide motifs in the normal and hypermutated HIV-1 sequences. Figure S2. Analysis of the hA3G and hA3F footprint on the negative strand of the HIV-1 sequences. Table S1. Details of the HIV-1 sequences identified as hypermutated at > 99.9% probability level using the proposed method in this paper. Figure S. The plot of DRhA3G versus DRhA3F for normal HIV-1 subtypes B, C and A1. [file 1742-4690-9-113-S1.docx]

**Additional file**

**Fig S1) DR_hA3G_ and DR_hA3F_ of preferred dimer, trimer and tetramer motifs in the normal and hypermutated HIV-1 sequences**

The p values show that the difference between normal and hA3G hypermutated sequences extends at least up to 4-mers. However in the case of sequences hypermutated by hA3F the difference is only evident for 2-mers.


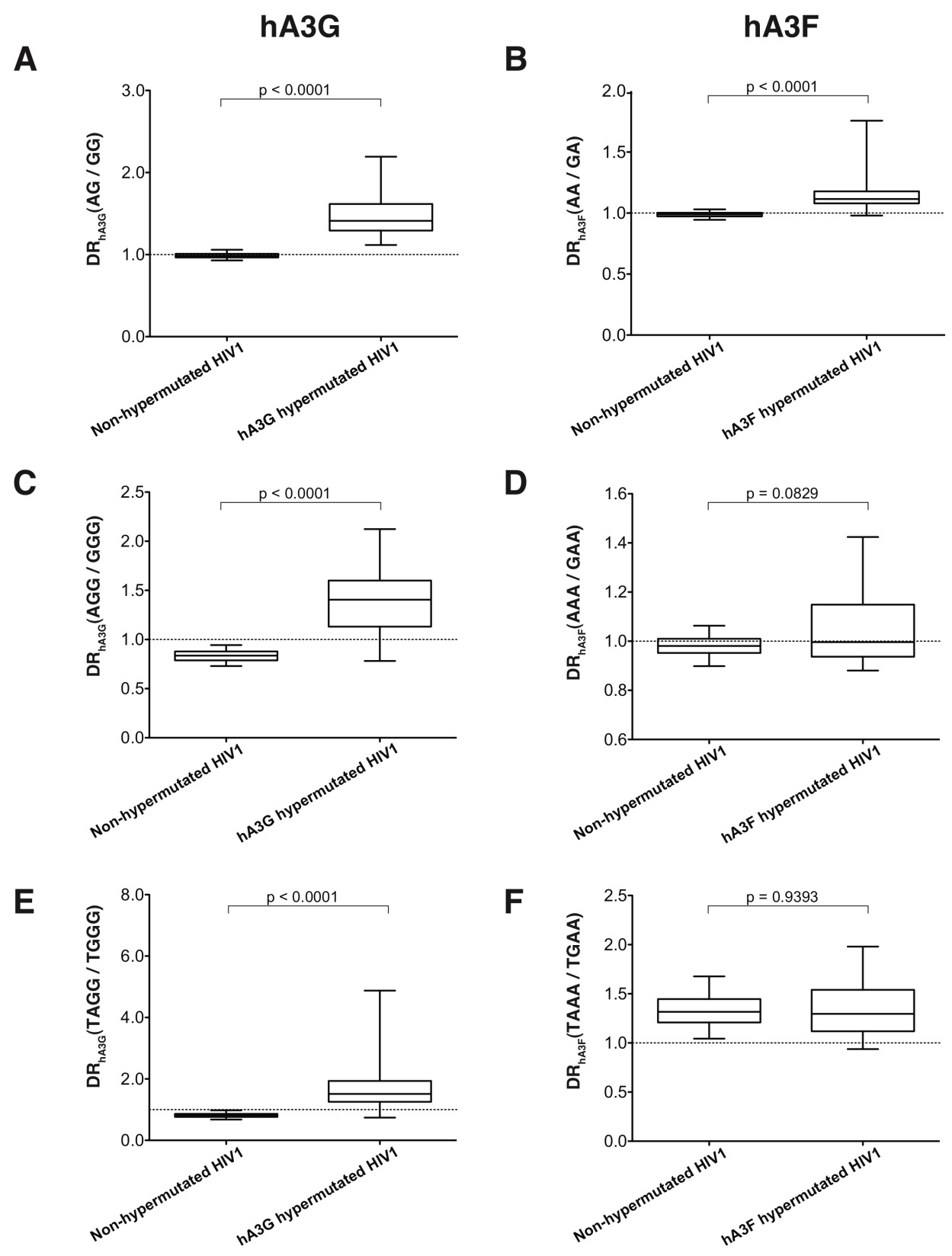


**Fig S2) Analysis of the hA3G and hA3F footprint on the negative strand of the HIV-1 sequences**

The hypermutation mechanism described in this paper leaves a G-to-A mutation footprint on the positive strand, but not on the negative strand. Therefore comparison of DRs of opposite strands can shed light on the hypermutation status of a given sequence. Fig S2 shows the DR_hA3G_ versus DR_hA3F_ values of the negative strands of the HIV-1 sequences used in Fig 2. Contrary to the pattern observed for the positive strand, here all the normal and hypermutated HIV-1 sequences form a tight cluster and do not extend in the direction of the DR_hA3G_ and DR_hA3F_ axes. In this figure the nominally normal and hypermutated sequences are shown by open black circles and open red triangles, respectively. The nominally normal sequences with *α*>>99.9% are shown by large filled circles. As expected the normal and hypermutated HIV sequences do not differ from one another in terms of DR_hA3G_ and DR_hA3F_ of the minus strand.

To investigate whether the misclassification of the sequences with α>>99.9% by the LANL database is due to the lack of a reference sequence to be compared against we further examined the source of these sequences. We found that for these sequences there is either no “accompanying” sequence with the same subtype in the database (sequences AF193275 and FJ388944) or the available sequences are from a different patient. For each of the latter cases we generated a consensus sequence using the reported sequences and used it in the Hypermut 2 program. The probability levels returned by Hypermut 2 for sequences FJ469751, JF683737, GU595150 and FJ388965 were 92% (n=90), 50% (n=13), 80% (n=14) and 70% (n=40), respectively. The numbers in bracket are the number of sequences used to generate the consensus sequence in each case. These low probability levels imply that Hypermut 2 fails to identify these hypermutated sequences, because the available consensus sequences do not represent correct ancestral sequences.

**Table S1.** Details of the HIV-1 sequences identified as hypermutated at > 99.9% probability level using the proposed method in this paper.

| Accession number | Group/ Subtype | Hotelling’s *T^2^* | DR_hA3F_ (AA/GA) | DR_hA3G_ (AG/GG) | Main source(s) of hypermutation |
| --- | --- | --- | --- | --- | --- |
| AF457057 | A1 | 4189.01 | 0.96 | 2.71 | hA3G |
| AF407419 | O | 1965.07 | 1.19 | 1.93 | hA3G and hA3F |
| JF689891 | B | 1850.30 | 0.90 | 2.19 | hA3G |
| JF689858 | B | 1664.81 | 0.86 | 2.17 | hA3G |
| EF165359 | C | 1655.65 | 0.89 | 2.14 | hA3G |
| AY945729 | 01_AE | 1575.78 | 1.76 | 0.80 | hA3F |
| AY561241 | B | 1565.56 | 0.95 | 2.06 | hA3G |
| EF165365 | A1 | 1318.31 | 1.01 | 1.92 | hA3G and hA3F |
| EF165363 | B | 1105.78 | 0.86 | 1.97 | hA3G |
| AY829213 | B | 1003.31 | 1.05 | 1.76 | hA3G and hA3F |
| GU201503 | 0102AG | 950.30 | 0.90 | 1.87 | hA3G |
| AY358055 | 01_AE | 924.93 | 0.88 | 1.88 | hA3G |
| DQ164125 | C | 898.26 | 0.98 | 1.78 | hA3G |
| JF689855 | B | 885.18 | 0.95 | 1.81 | hA3G |
| JF689881 | B | 841.58 | 0.88 | 1.84 | hA3G |
| GU201515 | 01_AE | 744.37 | 0.87 | 1.80 | hA3G |
| GU201517 | 02_AG | 683.93 | 1.50 | 0.85 | hA3F |
| EF165364 | D | 671.81 | 1.51 | 0.81 | hA3F |
| AY255828 | C | 588.23 | 0.91 | 1.69 | hA3G |
| JN251904 | BF | 572.28 | 0.93 | 1.67 | hA3G |
| JN029802 | - | 555.98 | 0.90 | 1.68 | hA3G |
| DQ164128 | C | 551.95 | 0.88 | 1.70 | hA3G |
| AY945714 | 01_AE | 522.92 | 0.90 | 1.66 | hA3G |
| DQ275665 | C | 484.11 | 0.93 | 1.62 | hA3G |
| AY945723 | 01_AE | 482.57 | 0.88 | 1.65 | hA3G |
| AY358058 | 01_AE | 480.58 | 0.94 | 1.61 | hA3G |
| GQ229530 | 22_01A1 | 467.85 | 0.94 | 1.60 | hA3G |
| AY945735 | 01C | 444.73 | 0.93 | 1.59 | hA3G |
| JF689888 | B | 437.44 | 0.92 | 1.60 | hA3G |
| JF689882 | B | 392.35 | 1.36 | 0.92 | hA3F |
| AF457074 | A1D | 381.54 | 0.91 | 1.57 | hA3G |
| AY734557 | C | 358.57 | 0.88 | 1.57 | hA3G |
| AY781125 | B | 338.98 | 0.96 | 1.50 | hA3G |
| JF689880 | B | 335.31 | 0.89 | 1.55 | hA3G |
| EF165366 | A1 | 322.20 | 0.92 | 1.52 | hA3G |
| EF165361 | 01_AE | 320.82 | 0.91 | 1.53 | hA3G |
| EF165360 | C | 307.93 | 0.91 | 1.51 | hA3G |
| AF457071 | A1 | 217.14 | 0.95 | 1.41 | hA3G |
| AF484484 | A1 | 216.62 | 0.90 | 1.45 | hA3G |
| AY037273 | BF | 206.27 | 0.89 | 1.44 | hA3G |
| AY358054 | 01_AE | 202.48 | 0.93 | 1.41 | hA3G |
| AF457076 | A1 | 196.00 | 0.90 | 1.42 | hA3G |
| JN235955 | BF | 183.61 | 0.90 | 1.41 | hA3G |
| AY945715 | 01_AE | 180.56 | 0.93 | 1.39 | hA3G |
| DQ164123 | C | 179.66 | 0.90 | 1.41 | hA3G |
| EF165362 | D | 174.16 | 0.91 | 1.40 | hA3G |
| DQ164124 | C | 161.87 | 0.91 | 1.39 | hA3G |
| GU201506 | 0102DF | 160.24 | 0.90 | 1.39 | hA3G |
| AF442568 | A1D | 156.77 | 0.91 | 1.38 | hA3G |
| GU564226 | 01_AE | 139.29 | 0.96 | 1.32 | hA3G |
| AF442567 | A1D | 136.09 | 0.93 | 1.34 | hA3G |
| AY237165 | 10A1 | 133.66 | 0.96 | 1.32 | hA3G |
| JN235961 | B | 130.99 | 0.91 | 1.35 | hA3G |
| JN248588 | 02_AG | 126.75 | 0.88 | 1.36 | hA3G |
| AY371148 | 0102DF | 121.61 | 0.92 | 1.33 | hA3G |
| AY037274 | B | 117.51 | 0.89 | 1.34 | hA3G |
| AY444810 | 02_AG | 101.66 | 0.92 | 1.31 | hA3G |
| JF689878 | B | 98.20 | 0.92 | 1.30 | hA3G |
| AY037276 | BF | 84.53 | 0.90 | 1.29 | hA3G |
| AY371152 | 211 | 74.88 | 1.18 | 0.86 | hA3F |
| FJ388897 | B | 74.50 | 0.97 | 1.23 | hA3G |
| GU201510 | 211 | 65.23 | 1.16 | 0.87 | hA3F |
| AY237166 | A1D | 57.69 | 0.95 | 1.22 | hA3G |
| AF193275 | A1 | 54.22 | 0.93 | 1.22 | hA3G |
| AY358053 | 01_AE | 48.23 | 0.93 | 1.21 | hA3G |
| EF178404 | B | 48.14 | 1.12 | 0.97 | hA3F |
| AF457060 | 16_A2D | 48.03 | 0.93 | 1.21 | hA3G |
| AY037279 | 12_BF | 47.95 | 0.94 | 1.21 | hA3G |
| FJ388900 | A1B | 43.46 | 1.11 | 0.97 | hA3F |
| AY371135 | 02_AG | 41.77 | 0.91 | 1.21 | hA3G |
| JF689861 | B | 41.76 | 0.92 | 1.20 | hA3G |
| GU201502 | 02_AG | 41.76 | 0.90 | 1.21 | hA3G |
| AF457091 | A1 | 29.86 | 0.96 | 1.15 | hA3G |
| FJ469751 | B | 27.75 | 0.91 | 1.17 | hA3G |
| FJ388922 | B | 27.31 | 1.08 | 0.99 | hA3F |
| FJ195087 | B | 23.28 | 0.95 | 1.14 | hA3G |
| FJ388944 | 02U | 21.44 | 1.09 | 0.88 | hA3F |
| AY779556 | B | 20.67 | 0.95 | 1.14 | hA3G |
| JF683737 | A1 | 20.17 | 1.07 | 0.84 | hA3F |
| AY773339 | D | 19.69 | 1.01 | 0.85 | hA3F |
| GU595150 | BF1 | 18.78 | 1.08 | 0.93 | hA3F |
| FJ388907 | A1 | 18.55 | 1.00 | 1.09 | hA3G |
| GU201507 | 0102AG | 18.34 | 0.97 | 1.12 | hA3G |
| FJ388965 | B | 17.94 | 1.08 | 0.89 | hA3F |
| AY531116 | B | 17.19 | 0.95 | 1.12 | hA3G |
| AY734561 | C | 15.42 | 0.94 | 1.12 | hA3G |

**Hypersign: a tool for identification of hypermutated sequences**

We have developed an made available an executable Java program called “Hypersign” for analysis of hypermutated sequences. The input of this program is one or more query HIV sequences that can be provided by the user as a .*fasta* file or can be pasted directly into the program. The output of this program for each query sequence is a confidence level associated with the Hotelling’s *T^2^* statistic of the sequence. This is the confidence level at which the null hypothesis (H0: The query sequence is not hypermutated) would be rejected. The other output of the software is a plot of DR_hA3G_ versus DR_hA3F_ from which one can determine the source and extent of hypermutation. We have also included in the program the data of four separate populations of subtypes A1, B, C and 01_AE for which there were enough sequences in the database to form a reasonable population. These data can be used to identify hypermutated sequences with a known subtype of A1, B, C or 01_AE. As depicted in Fig S3, sub-type specific populations form tighter clusters from which it might be easier to identify hypermutated sequences when the subtype of the hypermutated sequence is known.

**Fig. S3** The plot of DR_hA3G_ versus DR_hA3F_ for normal HIV-1 subtypes B, C and A1.

The broken lines show the 99.9% confidence intervals of the Hotelling’s *T^2^* statistic.
