# Supplementary figures and images for: APOBEC3G and APOBEC3F rarely co-mutate the same HIV genome
Source: Retrovirology. 2012 Dec 20;9:113. doi: 10.1186/1742-4690-9-113 (PMC3532371; doi:10.1186/1742-4690-9-113)

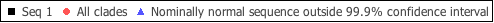

Supplement: Additional file 2 — Hypersign. A tool for identification of hypermutated sequences. [file 1742-4690-9-113-S2.zip › LegendTitleToImage.png]
